# Macon Medical Society

**Published:** 1885-11

**Authors:** 


### Society Reports.

### MACON MEDICAL SOCIETY.

Reported for THE ATLANTA MEDICAL AND SURGICAL JOURNAL.

STATED MEETING, August 4, 1885.

Dr. Ferguson in the chair.

The following voluntary paper was read by Dr. Ferguson :

### IS ANIMAL FOOD ESSENTIAL TO THE HUMAN ECONOMY ?

Many condemn without reflection new ideas advanced, because they do not emanate from acknowledged teachers, or because they are at variance with the accustomed dogmas, while the most complete absurdities uttered by authors of acknowledged repute are unhesitatingly gulped down for gospel truths. We are too prone to blindly follow in the footsteps of our predecessors, and take for granted the sum of their observations without exercising our own faculties to question the correctness of their views. I do not claim the views I set forth as wholly new, nor do I claim to be a pioneer in demonstrating the error of blindly following our ancestors in their false arguments, but I do claim that calm, unprejudiced reason and a careful comparison of the human race with other animals will teach us lessons of incalculable value in enabling us to live in accordance with nature's laws. In comparing the human structure with higher orders of the lower animal creation, we are forced to admit the homology that so clearly exists, and when we come to look at nature, untrammeled by the blood-hounds of civilization, unrestrained in the pursuit of health and pleasure, by customs of society, we feel our insignificance the more caustic as we pride ourselves on being the only animal endowed with reasoning powers. Upon a careful examination of the habits of those higher orders of the lower creation, as the quadrumana, we find these animals subsisting almost exclusively, with but few exceptions, on fruits, nuts, plants, bird's eggs, insects and young birds, and amongst the anthropomorphous apes we find some of these species even make a sort of shelter amongst the branches of trees. Examine the mouths of these animals and see what a close similarity in shape and arrangement of the teeth they bear to ours, with the exception in their favor for warranting them in eating animal food, for in all of those that indulge in animal food the canine teeth are developed to a degree far in excess of those of other species that confine themselves to fruits.

Compare the carnivora with man, and we find but an analogue of the most distinguishing characteristic of the former in the canine tooth of man, with no similarity whatever in the molars. Does this not show us most unerringly that we are not adapted to the indulgence of the excess of animal food we so habitually partake of. That animal food is truly pernicious to the human economy is daily being admitted. The beef essences, animal broths and soups, so highly lauded in former times in the treatment of debilitating diseases, are now pretty generally being abandoned for the more assimilable farinaceous articles so abundant in nature and readily prepared. Physicians have learned by hard experience that animal food does not afford the elements so requisite to sustain the vital forces through a long siege of fever, or any other enfeebling disease, as a vegetable diet which contains an abundant supply of nutrition at first cost, without having to undergo the many changes in digestion before being suited for assimilation.

Analysis shows flesh to contain from 70 to 74 per cent. of water, the dry residue being rich in nitrogen, and it contains a little carbonacious or fatty matter. Hence, to live on animal food alone, as much as eight pounds a day is necessary, and if even this allowance was adhered to for any great length of time, starvation would surely follow. That the minerals now indulged in as food are positively worthless to the human system has long since been proven by Banting, of England, who reduced himself from an object of disgusting obesity to the size of an ordinary man by the rigid and undeviating indulgence in animal food to the exclusion of all farinaceous articles. This fact is so well known among pugilists that, when wishing to reduce themselves from excessive weight and superabundance of adipose tissue to suitable proportions, beef constitutes their chief diet until that end is accomplished. Witness the cadaveric appearance of nearly all our farmers, whom we see daily on our streets, and whose chief diet consists of bacon and other animal food. Take the same class in Scotland, England, Ireland and Germany, whose chief diet consists almost exclusively of vegetables, and see the fine specimens of muscular development so universal in those nations. A discussion on vegetarianism took place at a meeting of the Medical Society of London, when Sir Joseph Fayrer related his experience of the effects of the diet among natives of India, and said he had no doubt that people could live on vegetables alone. He had seen some of the finest specimens of the human race, as regards strength, power of endurance and physical development, amongst the inhabitants of the northwest provinces of India, who were pure vegetarians. Witness the deterioration Europeans undergo after they land on the Continent and partake of the animal diet so constantly placed before them as food. How soon they lose the rotundity of form and healthful glow for the cadaveric form and swarthy skin of the average meat-eater. Compare the two classes. In the one, whose chief diet consists of vegetables, you have a hearty, robust, healthy-looking people ; in the orther, they look as if they had been dead, buried and dug up again. Do these facts not go to show that man can and must be sustained from the vegetable in place of the animal kingdom ? If the very animal food we eat is produced from the vegetable kingdom, why not we thrive equally on a vegetable diet ? It is a great mistake to say that animal food is muscle-producing, except amongst the carnivora, for if it was, why does it, when indulged in to the exclusion of all other articles, produce such fearful emaciation, and eventually starvation ? Aside from all this, there are the diseases incident to animal life transmissible through their flesh to human beings. I saw in Macon, a year or two ago, a kidney, of twice its normal size, taken from a cow that was slaughtered for market, having a number of cavities from the size of a pea to the diameter of an inch, containing spherical-shaped bodies from the size of a mustard seed to that of a marble, of a bluish clayey substance. I had no opportunity of making any analysis of the elementary constituents of these bodies. This beef was to all appearances good, healthy looking flesh, such as would be chosen by gourmands for a savory repast, and yet the animal could not have been healthy with such a diseased kidney, for to produce this diseased kidney some great derangement of the general health must have existed, and any general derangement must of necessity rendered the meat unfit for food. Now I do not intend to say that the disease with which this animal was afflicted was transmissible to those who ate the flesh, but I do say that such food must of necessity engender disease or some derangement of the system that would terminate in disease. In the vegetable kingdom any disease could readily be detected by the appearance of the plant or its obnoxious flavor when eaten. The long-continued use of animal food is productive of a disease that probably is unfamiliar to the majority of us, known as scurvy, and which was more frequently confined to sailors, whose diet consisted of salt meats, before canned fruits and vegetables were known. To restore the system to health from scurvy, a diet of vegetables was resorted to as the quickest, surest and best antidote. At the present time it is a disease seldom met with, and when found the same treatment is adopted, with the addition of large quantities of potash to add to the too meagre supply afforded by the vegetables for rapid restoration. I might go on and multiply any number of examples to show the ill effects of animal iood, but will rest with these already given as quite sufficient, and note the ill effects of vegetables on the carnivora. As a familiar example of the pernicious effects of vegetable food on the carnivora, witness the mangey curs on our streets.

Feed a dog or cat on animal food and no manifestation of skin disease will follow. They will always wear a healthy, sleek and natural appearance—be ever watchful and alive to all surroundings, but confine them to a vegetable diet, as most of our domestic carnivora are, and you have as the result not only a mangey animal, but one that is dull, stupid and unnatural. I firmly believe nine-tenths of the so-called mad-dogs are dogs diseased from eating food entirely foreign to the requirements of their systems and at a variance with natural laws. I have tested the matter out of curiosity, so far as to cure a dog of mange by a diet of raw beef, when all the so-called cures failed.

Place a diet of raw beef and one of vegetables, ever so enticingly cooked, together, and see which of the two a dog or cat will eat. The vegetables will remain untouched until decomposed as long as there is animal food to be eaten, and only when the pangs of hunger face the animal will it indulge in the vegetables.

The aggregate hours required to digest 27 articles of animal food in daily use is 87 hours. Of the same number of vegetables in daily use 74 hours, leaving 13 hours in favor of vegetables. In view of these facts, and the lack of animal diet supplying the elements requisite for the healthy nourishment of the system, it has long since been my custom to taboo the use of any flesh while my patients are under treatment for any disease. I am quite satisfied that by pursuing this course I have been able to combat and conduct to a successful end many cases that have heretofore resisted all other plans of treatment. In examining this subject, I have feebly tried to place some of my views in as few words as possible before the Society, with the hope that they may lead to profitable discussion, and draw attention to what I consider a very important matter, both in the treatment of disease and the preservation of health.

